# Evaluating methodological quality of prognostic prediction models on patient reported outcome measurements after total hip replacement and total knee replacement surgery: a systematic review protocol

**DOI:** 10.1186/s13643-022-02039-7

**Published:** 2022-08-10

**Authors:** Wei-Ju Chang, Justine Naylor, Pragadesh Natarajan, Victor Liu, Sam Adie

**Affiliations:** 1 Centre for Pain IMPACT, Neuroscience Research Australia (NeuRA), 139 Barker St, Randwick, NSW 2031 Australia; 2 School of Health Sciences, College of Health, Medicine and Wellbeing, The University of Newcastle, Callaghan, NSW 2038 Australia; 3 School of Clinical Medicine, UNSW Medicine & Health, South West Clinical Campuses, Discipline of Surgery, Faculty of Medicine and Health, UNSW, Sydney, NSW Australia; 4 Whitlam Orthopaedic Research Centre, Ingham Institute for Applied Medical Research, 1 Campbell St, Liverpool, NSW 2170 Australia; 5 St George and Sutherland Clinical School, University of New South Wales, Clinical Sciences (WRPitney) Building, Short Street, St George Hospital, Kogarah, NSW 2217 Australia; 6 St. George and Sutherland Centre for Clinical Orthopaedic Research (SCORe), Suite 201, Level 2 131 Princes Highway, Kogarah, NSW 2217 Australia; 7 School of Clinical Medicine, UNSW Medicine & Health, UNSW, New South Wales Sydney, Australia

**Keywords:** Total hip replacement, Total knee replacement, Prediction model, Systematic review, Meta-analysis

## Abstract

**Background:**

Prediction models for poor patient-reported surgical outcomes after total hip replacement (THR) and total knee replacement (TKR) may provide a method for improving appropriate surgical care for hip and knee osteoarthritis. There are concerns about methodological issues and the risk of bias of studies producing prediction models. A critical evaluation of the methodological quality of prediction modelling studies in THR and TKR is needed to ensure their clinical usefulness. This systematic review aims to (1) evaluate and report the quality of risk stratification and prediction modelling studies that predict patient-reported outcomes after THR and TKR; (2) identify areas of methodological deficit and provide recommendations for future research; and (3) synthesise the evidence on prediction models associated with post-operative patient-reported outcomes after THR and TKR surgeries.

**Methods:**

MEDLINE, EMBASE, and CINAHL electronic databases will be searched to identify relevant studies. Title and abstract and full-text screening will be performed by two independent reviewers. We will include (1) prediction model development studies without external validation; (2) prediction model development studies with external validation of independent data; (3) external model validation studies; and (4) studies updating a previously developed prediction model. Data extraction spreadsheets will be developed based on the CHARMS checklist and TRIPOD statement and piloted on two relevant studies. Study quality and risk of bias will be assessed using the PROBAST tool. Prediction models will be summarised qualitatively. Meta-analyses on the predictive performance of included models will be conducted if appropriate. A narrative review will be used to synthesis the evidence if there are insufficient data to perform meta-analyses.

**Discussion:**

This systematic review will evaluate the methodological quality and usefulness of prediction models for poor outcomes after THR or TKR. This information is essential to provide evidence-based healthcare for end-stage hip and knee osteoarthritis. Findings of this review will contribute to the identification of key areas for improvement in conducting prognostic research in this field and facilitate the progress in evidence-based tailored treatments for hip and knee osteoarthritis.

**Systematic review registration:**

PROSPERO registration number CRD42021271828.

**Supplementary Information:**

The online version contains supplementary material available at 10.1186/s13643-022-02039-7.

## Background

Osteoarthritis affects 9% of the population and over 30% of those aged > 65 years in Australia, cost the health care system an estimated $3.5 billion in 2015–2016 [1]. Total hip and knee replacement (THR and TKR) surgeries are effective for treating end-stage hip and knee osteoarthritis [2]. However, some patients report unsatisfactory outcomes persistent pain or poor function following THR (~ 5–10%) and TKR (~ 15–35%) [3–5]. Unsatisfactory surgical outcomes may lead to revision and a further increase in healthcare burdens [6, 7]. Identifying individuals who may not respond to THR or TKR can assist the development of new non-operative treatment strategies for this subgroup, ensuring surgery is only provided to those most likely to benefit. However, inappropriate use of prediction models could potentially deny THR or TKR to patients who could benefit from surgery. Thus, the potential impact of these prediction models on osteoarthritis populations is substantial. Well-constructed prediction models that can predict poor patient-reported surgical outcomes are crucial to inform clinical decision making. Furthermore, a critical evaluation of the methodological quality of prediction modelling studies in THR and TKR is needed to ensure their clinical usefulness.

The reporting quality of research aimed to develop or validate prediction models is considered suboptimal in medicine and significant efforts have been made to improve methodological rigor and research transparency in this field [8]. Guidelines and instruments such as the TRIPOD Statement [9] and CHARMS checklist [10] have been developed to provide guidance for reporting prognostic studies. Indeed, systematic reviews on the methodological quality of prognostic models have been performed in conditions such as hypertension [11], chronic kidney disease [12, 13], and cancer [14], but are absent in joint replacement, despite much attention directed at the patient selection and optimisation of joint replacement [15]. Thus, little is known about the quantity, validity and methodological quality of studies that have generated prediction models for specific outcomes after THR and TKR.

Published prognostic models in total joint replacement range from predicting perioperative complications [16–18] and discharge destination [19], to long-term postoperative outcomes including pain [20, 21], infection [22–25], readmission [26], revision [27], patient-reported function [28, 29], range of motion [30], and satisfaction [31]. Although studies have identified risk factors (e.g. psychological distress, diabetes and severe obesity) [32–36] and prediction models for poor patient-reported outcome after joint replacement [20, 21, 28, 29], there are concerns about methodological issues and the risk of bias [37, 38]. The lack of methodological and analytic rigour of these studies indicates the risk that incorrect models could be used to guide clinical practice.

This systematic review aims to (1) evaluate and report the quality of risk stratification and prediction modelling studies that predict patient-reported outcomes after THR and TKR; (2) identify areas of methodological deficit and provide recommendations for future research; and (3) synthesise the evidence on prediction models associated with post-operative patient-reported outcomes after THR and TKR surgery.

## Methods/design

This systematic review protocol is prepared according to the Preferred Reporting Items for Systematic Reviews and Meta-Analysis Protocols (PRISMA-P) Guidelines [39, 40]. The PRISMA-P checklist is provided in the Supplementary Files (Table S1). This systematic review has been registered with the International Prospective Register of Systematic Reviews (PROSPERO, registration number CRD42021271828).

### Eligibility criteria

The PICOTS (Population, Intervention, Comparison, Outcome, Timing, Setting) approach is used to develop the eligibility criteria that will be used to select relevant studies [9, 41]. This information is provided in the Supplementary Files (Table S2).

### Types of participants

Studies including adults aged 18 or older receiving elective THR or TKR will be included. The surgeries can be either primary or revision for persistent pain after previous THR/TKR, and either unilateral or bilateral joint replacement. No restriction will be placed on sex or race. Studies including participants receiving megaprosthesis for sarcoma, partial/hemi-replacements, or THR/TKR indicated for acute fracture, will be excluded.

### Types of studies

We will evaluate prospective studies using multivariate predictive statistical models that assess preoperative risk factors for predicting patient-reported outcome after THR or TKR. We will include the following studies:

1. Prediction model development studies without external validation of independent data.
2. Prediction model development studies with external validation of independent data.
3. External model validation studies or temporal validation studies.
4. Studies updating a previously developed prediction model.

Eligible studies should present at least one formal prediction model or regression equation in such a way that it allows calculation of the risk of poor post-operative outcome defined by the study authors.

Included studies must have patient-reported outcome measures (PROMs) as the primary prediction outcome. As there is no single validated, reliable and responsive PROM specifically for TKR or THR, we will include prediction models using instruments to measure minimally clinically important difference in any patient-reported outcomes [42]. These instruments include generic (quality of life) questionnaires such as the Short Form health surveys (SF-36 or SF-12) and the EuroQol 5-dimension questionnaire, or joint-specific questionnaires such as the Knee Society Score, the Western Ontario and McMaster Universities Arthritis Index, Oxford Knee/Hip Score or Hip disability, and Osteoarthritis Outcome Score [43]. Although studies may investigate models including pre-, peri- or post-operative predictor variables, eligible studies should report a final prediction model(s) that only includes pre-operative predictor variables.

The following types of study will be excluded:

1. Univariate prediction studies reporting bivariate associations between specific baseline clinical risk factors and postoperative PROMs, without multivariate adjustment for other sociodemographic or clinical parameters.
2. Studies only identifying predictors associated with a PROM without an attempt to develop a prediction model.
3. Studies that only predict non-PROM postoperative outcomes such as adverse events, complication rates, revision, falls, or clinician assessed/reported outcomes.
4. Literature reviews and grey literature such as reports, conference abstracts, opinions, editorials, commentaries, letters. However, the reference lists of literature review will be screened for potentially relevant studies.

### Search strategy

To identify relevant studies, an electronic literature search of MEDLINE, EMBASE, and CINAHL will be conducted. Available published search filters will be adapted and combined with medical subject headings (MeSH) and related free-text words for a sensitive yet specific search strategy. A combination of different keywords for THR or TKR and prediction model will be used to identify relevant literature. The search strategies will be tailored to each database. The full search terms and search strategy are included in the Supplementary Files (Table S3). No restriction will be placed on the publication period. Only articles in the English language will be included. If non-English studies have English abstract, they will be included in the title and abstract screening, but excluded from the full-text screening. The reference lists of included studies and existing relevant reviews will also be screened for potentially relevant studies. References will be searched for the original prediction model development study in cases of external model updating and recalibration. While the review is in progress, citation searching for forward citation of recent studies and citation alerts (e.g. Google Scholar) will be used to identify potentially relevant studies as they appear. The searches will be re-run prior to the final analysis and new relevant studies will be retrieved.

### Study selection

The complete references of the studies retrieved from the above search strategy will be imported into Endnote X9 and duplicates removed. Two reviewers will independently assess the title and abstract of all studies identified through the search against the eligibility criteria. The full text of all eligible studies will then be retrieved. Disagreements on study eligibility will be resolved by consensus and if necessary, a third reviewer will be consulted for arbitration. Search results and reasons for excluded articles at each stage of study selection will be documented and reported in a PRISMA flowchart [44].

### Data extraction

Two reviewers will independently conduct the data extraction from the final list of eligible studies. Any disagreements in the extracted data will be resolved through discussion with an additional reviewer. A piloting phase will be introduced before the formal data extraction. During the piloting phase, two randomly chosen articles from the eligible articles will be used by two independent reviewers to test a piloted data extraction spreadsheet and the definitions of the items to be collected. Disagreements will be discussed to achieve consensus and modifications to the piloted spreadsheet will be made. This customised data extraction spreadsheet will be reviewed and agreed by all the reviewers before its use in the formal data extraction. The agreement between two reviewers for risk of bias assessment and data extraction will be assessed using Kappa statistics.

We will collect information in the domains related to prediction modelling adapted from the CHARMS (CHecklist for critical Appraisal and data extraction for systematic Reviews of prediction Modelling Studies) and TRIPOD (Transparent Reporting of a multivariable prediction model for Individual Prognosis Or Diagnosis) statements [10, 45]. The following information will be extracted from the eligible studies:

- *Study characteristics—*first author, publication year, data source (cohort, case-control, randomised trial participants, registry data, electronic medical record data or separate development dataset), study dates (start and end of accrual, end of follow-up), recruitment method.
- *Participants—*age, sex, type of surgery, the number of participants enrolled in the study.
- *Outcome measures—*defined outcome of interest (PROMs such as pain, function, mobility, composite outcome), method of outcome measurement, where the same definition and method used for all participants (Y/N), type of outcome (single, combined endpoints), blinding of outcomes assessors (Y/N), candidate predictors part of outcome in panel or consensus diagnosis (Y/N), time duration of outcome occurrence.
- *Predictor variables—*type of predictors, number of predictors included in final model, defined method for measurement of candidate predictors (Y/N), timing of predictor measurement, blinding of predictor assessment including blind for outcome and blind for each other (Y/N), handling of predictors in modelling.
- *Model sample size—*number of participants, number of outcome events reported, events per variable, number of outcomes in relation to number of predictor variables.
- *Missing data—*number of participants with missing data in each predictor variables and outcome measures, handling of missing data.
- *Model development—*modelling method, modelling assumptions satisfied (Y/N), predictor pre-selection for inclusion in multivariate modelling, predictor selection method during multivariate modelling, criteria for predictor selection.
- *Model performance—*calibration, discrimination, whether performance measures with confidence intervals (Y/N). Prediction model performance including *discrimination* using a c-statistic such as area under the receiver operating characteristic (ROC) curve (AUC), *calibration* using a calibration plot and slope or goodness-of-fit statistic (e.g. Hosmer-Lemeshow test), or overall model fit (e.g. Brier score, explained variation/*R*^2^ statistic) will be extracted. Further, if a decision curve analysis was conducted in the studies, findings of such analysis (e.g. net benefit) will also be extracted.
- *Model performance evaluation—*internal and/or external validation methods, was there poor validation with model testing (Y/N) including model adjusted/updated (Y/N), adjustment such as intercept recalibrated, predictor effects adjusted, or new predictors added (Y/N).
- *Result-* final multivariable models, alternative presentation of final prediction models (Y/N), comparison of predictors distribution (including missing data) for development and validation datasets (Y/N).

When data are missing, authors of the studies will be contacted a maximum of three times in order to obtain the data.

### Quantitative data extraction and pre-processing

Discrimination is the ability of a prediction model to differentiate between participants who develop poor outcome and those who do not, assessed using c-statistics (such as AUC). C-statistics with 95% confidence intervals will be extracted. As the discrimination of prediction models is heavily influenced by the distribution of participant characteristics, or case mix variation, the standard deviation of participant characteristics (e.g. age) and of the linear predictor for the outcome of interest will be extracted [41]. The linear predictor is defined as the weighted sum of the values of predictors in the validation study, where the weights are the regression coefficients of the prediction model [41, 46]. When the standard deviation is unavailable, reported ranges will be used to obtain such information [41].

Calibration is the agreement between outcome predicted by the model and the observed outcome [47]. The calibration slope of the calibration plot, if reported, will be extracted and summarised. However, as calibration is often reported using different summary statistics or unreported, the total number of observed (O) and expected (E) events will be extracted and the total O:E ratio will be calculated to estimate the overall model calibration [47]. Where the O:E ratio is available in subgroups, such information will be extracted.

### Study quality and risk of bias

The methodological quality of the included studies will be assessed by two reviewers independently with disagreements resolved by consensus. The risk of bias and applicability concerns will be assessed using the PROBAST (Prediction model Risk Of Bias ASsessment Tool) [48] in four domains of participants, predictors, outcome, and analysis (a total of 20 signalling questions) for the development and validation of prediction models. These criteria are summarised in the Supplementary Files. Signalling questions will be rated (yes, probably yes, probably no, no or no information) to help make judgement for risk of bias as “high,” “low” or “unclear” for each domain. Applicability concerns of three domains of participants, predictors and outcome will also be rated (high/low/unclear). Overall risk of bias for each prediction model will be assessed across all four domains based on the following criteria:

- *Low—*all domains rated as low risk of bias; a prediction development model without external validation based on a very large data set and included internal validation.
- *High—*at least one domain rated as high risk of bias; a prediction development model without internal or external validation rated as low risk of bias.
- *Unclear—*at least one domain rated as unclear risk of bias and rest of the other domains as low risk of bias.

Overall applicability concerns for each model will be assessed across three domains according to the following criteria:

- *Low—*all domains rated as low concerns about applicability.
- *High—*at least one domain rated as high concerns about applicability.
- *Unclear—*at least one domain rated as unclear concerns about applicability.

If studies assessed multiple prediction models, only models meeting the eligibility criteria will be assessed for their risk of bias and applicability concerns.

### Data synthesis

#### Narrative review

A narrative review will be conducted to synthesise the evidence for the risk of bias and applicability concerns of the prediction modelling studies. Data of the selected studies will be tabulated or categorised in the following domains:

- *Study characteristics—*first author, publication year, study country, recruitment period, type of surgery, outcome measures, data source, age/sex of participants, number of participants included in derivation cohort/analysis for model development.
- *Outcomes—*type of PROMs, incidence of poor outcome (number and percentage).
- *Predictors for each outcome—*demographic, biological, psychological predictors.
- *Methodological findings—*model type, predictor selection procedure, predictor variables included in the model, missing data handling.
- *Model performance for each outcome—*predictive performance of development model (discrimination and calibration), type of validation, predictive performance of validation model.
- *Methodological quality—*risk of bias, applicability concerns.

All issues related to methodological quality will be reported and discussed. Specifically, the usefulness and overall applicability of the prediction models will be described. Findings will be presented based on the type of surgeries (THR vs. TKR), type of outcome predicted in the studies (e.g. pain, function, quality of life, composite measure) and type of model (e.g. logistic regression vs. machine learning). The risk of bias and applicability concerns will be reported as counts and percentages to underline the most critically affected domains of bias and applicability.

#### Meta-analysis

Quantitative analysis of this review will be conducted using R, version 4.03 (R Development Core Team, Vienna, Austria) [49] and relevant packages (e.g. ‘*metafor*’). Meta-analysis for measures of model performance will be conducted separately for the intervention (first THR and TKR, then primary and revision surgery if there are sufficient studies) and PROMs. When there are at least two included studies that assessed the prediction performance (discrimination and calibration) of the models on the same PROM with sufficient information available, meta-analysis will be performed to estimate the average model performance using a random effects model where the weights are based on the within-study error variance [41]. Estimates of discrimination and calibration will be first summarised separately. A joined synthesis of discrimination and calibration will then be performed using multivariate meta-analysis to avoid excluding studies that only assessed one of the measures of prediction performance [50]. Forest plots and hierarchical summary receiver operating characteristic (HSROC) curves will be produced to visualise model performance.

To assess the heterogeneity of the study population, Cochran’s *Q* and the I^2^ statistic will be calculated [51]. The heterogeneity is considered significant when *p* < 0.1 and *I*^2^ ≥ 50%. Difference between the 95% confidence intervals and prediction region in the HSROC curve will be used to visualise the heterogeneity, with a large difference indicating the presence of heterogeneity [52]. If more than 10 studies are included in the meta-analysis, sources of heterogeneity will be examined using meta-regression, where the dependent variable is the measure of model performance and the study level or summarised participant level characteristics (e.g. age) are the independent variables [41].

#### Subgroup and sensitivity analysis

Where heterogeneity is identified (*p* < 0.1), subgroup analysis will be performed based on type of model validation (internal and external validation), predictor variable selection method (forward or backward stepwise approaches, least absolute shrinkage and selection operator [LASSO] technique) and type of predictor variables selected in the models (clinical measures and laboratory-based measures) and other study characteristics according to the data extracted. A sensitivity analysis will be conducted to assess the impact of excluding studies with high risk of bias determined using the PROBAST tool, and the influence of type of arthroplasty (primary vs. revision) if data allow for such analysis.

#### Meta-biases

Publication biases will be assessed using a funnel plot to evaluate publication bias if more than 10 studies are included in meta-analysis [53]. Egger’s test will be used to assess the publication bias (*p* value > 0.10 indicating low publication bias), and a funnel plot asymmetry test will be conducted to examine the risk of publication bias (*p* value > 0.10 indicated low publication bias) [54]. A trim and fill method, a non-parametric data augmentation approach, will be used to estimate the number of missing studies and to generate an adjusted estimate by imputing suspected missing studies [55]. The adjusted estimates reveal whether the estimates based on meta-analysis are biased resulted from funnel plot asymmetry. If the difference between unadjusted and adjusted estimates is a positive value, the estimate in meta-analysis is considered overestimated due to missing studies [56].

### Reporting and dissemination

Findings from this review will be reported according to the Preferred Reporting Items for Systematic Reviews and Meta-Analysis (PRISMA) 2020 statement [57] and the confidence of evidence will be assessed using the Grades of Recommendation, Assessment, Development, and Evaluation (GRADE) system [58]. Any deviation from the protocol will be recorded and explained in the final report. We will disseminate our findings in published in peer-reviewed journals and presented at national/international conferences related to orthopaedic medicine.

## Discussion

This protocol describes a systematic review to evaluate the methodological quality and the usefulness of prediction models for poor patient-reported outcomes after THR or TKR. This information is essential to provide evidence-based recommendations for clinical decision making in healthcare for individuals with end-stage hip and knee osteoarthritis. Well-conducted prediction modelling studies have great potential to inform research and clinical practice in stratified treatments based on accurate risk estimates. Findings of this review will contribute to the identification of key areas for improvement in conducting prognostic research in this field and facilitate the progress in evidence-based tailored treatments for hip and knee osteoarthritis.

## Supplementary Information

**Additional file 1: Supplementary Table S1.** PRISMA-P 2015 Checklist. **Supplementary Table S2.** Eligibility criteria framed using the PICOTS approach. **Supplementary Table S3.** Search strategies for electronic databases. **Supplementary Table S4.** PROBAST- Prediction model Risk Of Bias ASsessment Tool.

## Data Availability

Not applicable.

## Abbreviations

AUC: Area under the receiver operating characteristics curve
CHARMS: CHecklist for critical Appraisal and data extraction for systematic Reviews of prediction Modelling Studies
GRADE: Grades of Recommendation, Assessment, Development, and Evaluation
HSROC: Hierarchical summary receiver operating characteristic
LASSO: Least absolute shrinkage and selection operator
MeSH: Medical subject headings
PICOTS: Population, Intervention, Comparison, Outcome, Timing, Setting
PRISMA: Preferred Reporting Items for Systematic Reviews and Meta-Analysis
PRISMA-P: Preferred Reporting Items for Systematic Reviews and Meta-Analysis Protocols
PROBAST: Prediction model Risk Of Bias ASsessment Tool
PROM: Patient-reported outcome measure
PROSPERO: International Prospective Register of Systematic Reviews
ROC: Receiver operating characteristic
THR: Total hip replacement
TKR: Total knee replacement
TRIPOD: Transparent Reporting of a multivariable prediction model for Individual Prognosis Or Diagnosis
