# Supplementary material for: Evaluating methodological quality of prognostic prediction models on patient reported outcome measurements after total hip replacement and total knee replacement surgery: a systematic review protocol
Source: Syst Rev. 2022 Aug 10;11:165. doi: 10.1186/s13643-022-02039-7 (PMC9364604; doi:10.1186/s13643-022-02039-7)
Supplement: Supplementary file 1 — Additional file 1: Supplementary Table S1. PRISMA-P 2015 Checklist. Supplementary Table S2. Eligibility criteria framed using the PICOTS approach. Supplementary Table S3. Search strategies for electronic databases. Supplementary Table S4. PROBAST- Prediction model Risk Of Bias ASsessment Tool. [file 13643_2022_2039_MOESM1_ESM.docx]

**Supplementary Table S1.** **PRISMA-P 2015 Checklist**

| **Section/topic** | **#** | **Checklist item** | **Information reported** | | **Page number(s)** |
| --- | --- | --- | --- | --- | --- |
|  |  |  | **Yes** | **No** |  |
| **ADMINISTRATIVE INFORMATION** | | | | | |
| **Title** | | | | | |
| Identification | 1a | Identify the report as a protocol of a systematic review |  |  | 1 |
| Update | 1b | If the protocol is for an update of a previous systematic review, identify as such |  |  | Not applicable |
| **Registration** | 2 | If registered, provide the name of the registry (e.g., PROSPERO) and registration number in the Abstract |  |  | 3 |
| **Authors** | | | | | |
| Contact | 3a | Provide name, institutional affiliation, and e-mail address of all protocol authors; provide physical mailing address of corresponding author |  |  | 1 |
| Contributions | 3b | Describe contributions of protocol authors and identify the guarantor of the review |  |  | 17 |
| **Amendments** | 4 | If the protocol represents an amendment of a previously completed or published protocol, identify as such and list changes; otherwise, state plan for documenting important protocol amendments |  |  | Not applicable |
| **Support** | | | | | |
| Sources | 5a | Indicate sources of financial or other support for the review |  |  | 17 |
| Sponsor | 5b | Provide name for the review funder and/or sponsor |  |  | Not applicable |
| Role of sponsor/funder | 5c | Describe roles of funder(s), sponsor(s), and/or institution(s), if any, in developing the protocol |  |  | Not applicable |
| **INTRODUCTION** | | | | | |
| **Rationale** | 6 | Describe the rationale for the review in the context of what is already known |  |  | 4-5 |
| **Objectives** | 7 | Provide an explicit statement of the question(s) the review will address with reference to participants, interventions, comparators, and outcomes (PICO) |  |  | 5 |
| **METHODS** | | | | | |
| **Eligibility criteria** | 8 | Specify the study characteristics (e.g., PICO, study design, setting, time frame) and report characteristics (e.g., years considered, language, publication status) to be used as criteria for eligibility for the review |  |  | 6-7 |
| **Information sources** | 9 | Describe all intended information sources (e.g., electronic databases, contact with study authors, trial registers, or other grey literature sources) with planned dates of coverage |  | 8 | 7-8 |
| **Search strategy** | 10 | Present draft of search strategy to be used for at least one electronic database, including planned limits, such that it could be repeated |  |  | Supplementary files page 5 |
| ***STUDY RECORDS*** | | | | | |
| Data management | 11a | Describe the mechanism(s) that will be used to manage records and data throughout the review |  |  | 8 |
| Selection process | 11b | State the process that will be used for selecting studies (e.g., two independent reviewers) through each phase of the review (i.e., screening, eligibility, and inclusion in meta-analysis) |  |  | 8 |
| Data collection process | 11c | Describe planned method of extracting data from reports (e.g., piloting forms, done independently, in duplicate), any processes for obtaining and confirming data from investigators |  |  | 8-9 |
| **Data items** | 12 | List and define all variables for which data will be sought (e.g., PICO items, funding sources), any pre-planned data assumptions and simplifications |  |  | 9-10 |
| **Outcomes and prioritization** | 13 | List and define all outcomes for which data will be sought, including prioritization of main and additional outcomes, with rationale |  |  | 9-10 |
| **Risk of bias in individual studies** | 14 | Describe anticipated methods for assessing risk of bias of individual studies, including whether this will be done at the outcome or study level, or both; state how this information will be used in data synthesis |  |  | 11-12 |
| ***DATA*** | | | | | |
| **Synthesis** | 15a | Describe criteria under which study data will be quantitatively synthesized |  |  | 13 |
|  | 15b | If data are appropriate for quantitative synthesis, describe planned summary measures, methods of handling data, and methods of combining data from studies, including any planned exploration of consistency (e.g., *I* ^2^, Kendall’s tau) |  |  | 13-14 |
|  | 15c | Describe any proposed additional analyses (e.g., sensitivity or subgroup analyses, meta-regression) |  |  | 14 |
|  | 15d | If quantitative synthesis is not appropriate, describe the type of summary planned |  |  | 12-13 |
| **Meta-bias(es)** | 16 | Specify any planned assessment of meta-bias(es) (e.g., publication bias across studies, selective reporting within studies) |  |  | 14-15 |
| **Confidence in cumulative evidence** | 17 | Describe how the strength of the body of evidence will be assessed (e.g., GRADE) |  |  | Not applicable |

**Supplementary Table S2. Eligibility criteria framed using the PICOTS approach.**

| **PICOTS approach** | **Inclusion** | **Exclusion** | **Consideration** |
| --- | --- | --- | --- |
| ***Prediction modelling for patient-reported outcomes in individuals intend to receive THR*** | | | |
| *Population* | Persistent hip osteoarthritis patient (≥ 18 years old) who plan to receive THR | Individuals < 18 years old; undiagnosed hip pain patients, general population without hip pain | Studies included people with hip osteoarthritis diagnosed by any criteria planning to receive THR will be considered for inclusion. Studies included people intend to undergo revision THR for persistent pain after previous THR will also be included. |
| *Index* | Development and/or validation of a prediction model for individuals who plan to receive THR to predict patient-reported outcomes | Development and/or validation of a prediction model for individuals who plan to receive THR to predict THR-related complications; retrospective studies investigate prognostic risk factors of patient-reported outcomes or complications after THR | Prediction model development with or without validation, and validation with or without updating will be considered for inclusion, if they aimed to predict patient-reported outcomes after THR. |
| *Comparator* | Not applicable | Not applicable | As there has been no widely accepted prediction prediction model for patient-reported outcomes after THR based on pre-operative factors, a comparison is unlikely to be conducted. |
| *Outcomes* | Primary outcome: post-THR patient-reported outcomes such as pain, function, quality of life, patient satisfaction. | Objective outcome measures; outcomes measured immediately after the surgery (peri-operative outcomes); outcome measures of re-operation rate, complications etc; outcomes or complications unrelated to THR | We are only interested in prediction models aimed to identify individuals who are unlikely benefit from THR, thus only patient-reported outcome measures are considered informative for this review. |
| *Timing* | Patient-related outcomes measured after THR | Peri-operative factors as predictors will be excluded | We are only interested in prediction models based on pre-operative factors. |
| *Setting* | Pre-operative use for healthcare professionals in orthopaedics or general practice setting | Prediction models to be used peri- or post-THR | Prediction models aim to inform clinical decision making for selecting suitable patients for THR or identifying patient with high risk to develop poor outcomes. |
| ***Prediction modelling for patient-reported outcomes in individuals intend to receive TKR*** | | | |
| *Population* | Persistent knee osteoarthritis patient (≥ 18 years old) who plan to receive TKR | Individuals < 18 years old; undiagnosed knee pain patients, general population without knee pain | Studies included people with knee osteoarthritis diagnosed by any criteria planning to receive TKR will be considered for inclusion. Studies included people intend to undergo revision TKR for persistent pain after previous TKR will also be included. |
| *Index* | Development and/or validation of a prediction model for individuals who plan to receive TKR to predict patient-reported outcomes | Development and/or validation of a prediction model for individuals who plan to receive TKR to predict TKR-related complications; retrospective studies investigate prognostic risk factors of patient-reported outcomes or complications after TKR | Prediction model development with or without validation, and validation with or without updating will be considered for inclusion, if they aimed to predict patient-reported outcomes after TKR. |
| *Comparator* | Not applicable | Not applicable | As there has been no widely accepted prediction model for patient-reported outcomes after TKR based on pre-operative factors, a comparison is unlikely to be conducted. |
| *Outcomes* | Primary outcome: post-TKR patient-reported outcomes such as pain, function, quality of life, patient satisfaction. | Objective outcome measures; outcomes measured immediately after the surgery (peri-operative outcomes); outcome measures of re-operation rate, complications etc; outcomes or complications unrelated to TKR | We are only interested in prediction models aimed to identify individuals who are unlikely benefit from TKR, thus only patient-reported outcome measures are considered informative for this review. |
| *Timing* | Patient-related outcomes measured after TKR | Peri-operative factors as predictors will be excluded | We are only interested in prediction models based on pre-operative factors. |
| *Setting* | Pre-operative use for healthcare professionals in orthopaedics or general practice setting | Prediction models to be used peri- or post-TKR | Prediction models aim to inform clinical decision making for selecting suitable patients for TKR or identifying patient with high risk to develop poor outcomes. |

Note: THR- total hip replacement; TKR- total knee replacement.

**Supplementary Table S3. Search strategies for electronic databases.**

| **Database** | **Search strategy** |
| --- | --- |
| Medline/PubMed | #1 'knee'/exp OR 'knee':ab,ti OR 'hip'/exp OR 'hip':ab,ti  #2 'osteoarthritis'/exp OR 'osteoarthritis':ab,ti OR 'osteoarthrosis':ab,ti OR 'degenerative arthritis':ab,ti OR osteoarthr*:ab,ti OR "OA":ab,ti  #3 'knee oa':ab,ti OR 'KOA':ab,ti OR 'hip oa':ab,ti  #4 (arthroplast* OR replace* OR prosthes*) OR (“Arthroplasty, Replacement, Hip”[Mesh] OR “Arthroplasty, Replacement, Knee”[Mesh]))  #5 'prediction model':ab,ti OR predict*:ab,ti OR progn*:ab,ti OR 'risk prediction':ab,ti OR 'risk score':ab,ti OR 'risk calculation':ab,ti OR 'risk assessment':ab,ti OR 'c statistic':ab,ti OR 'discrimination':ab,ti OR 'calibration':ab,ti OR 'auc':ab,ti OR 'area under the curve':ab,ti OR 'area under the receiver operator characteristic curve':ab,ti  #6 (((#1 AND #2) OR #3) AND #4) and #5 |
| Embase | #1 'knee'/exp OR 'knee':ab,ti OR 'hip'/exp OR 'hip':ab,ti  #2 'osteoarthritis'/exp OR 'osteoarthritis':ab,ti OR 'osteoarthrosis':ab,ti OR 'degenerative arthritis':ab,ti OR osteoarthr*:ab,ti OR "OA":ab,ti  #3 'knee oa':ab,ti OR 'KOA':ab,ti OR 'hip oa':ab,ti  #4 arthroplast*:ab,ti OR replace*:ab,ti OR prosthes*:ab,ti OR 'arthroplasty':ab,ti OR 'replacement':ab,ti  #5 'prediction model':ab,ti OR predict*:ab,ti OR progn*:ab,ti OR 'risk prediction':ab,ti OR 'risk score':ab,ti OR 'risk calculation':ab,ti OR 'risk assessment':ab,ti OR 'c statistic':ab,ti OR 'discrimination':ab,ti OR 'calibration':ab,ti OR 'auc':ab,ti OR 'area under the curve':ab,ti OR 'area under the receiver operator characteristic curve':ab,ti  #6 (((#1 AND #2) OR #3) AND #4) and #5 |
| CINAHL | #1 (TI knee OR AB knee OR SU knee) OR (TI hip OR AB hip OR SU hip)  #2 (TI osteoarthritis OR AB osteoarthritis OR SU osteoarthritis) OR (TI osteoarthrosis OR AB osteoarthrosis OR SU osteoarthrosis) OR (TI “degenerative arthritis” OR AB “degenerative arthritis” OR SU “degenerative arthritis”) OR (TI osteoarthr* OR AB osteoarthr* OR SU osteoarthr*) OR (TI OA OR AB OA OR SU OA)  #3 ((TI “knee OA” OR AB “knee OA” OR SU “knee OA”) OR (TI KOA OR AB KOA OR SU KOA)) OR ((TI “hip OA” OR AB “hip OA” OR SU “hip OA”))  #4 (TI arthroplasty OR AB arthroplasty OR SU arthroplasty) OR (TI replacement OR AB replacement OR SU replacement) OR (TI arthroplast* OR AB arthroplast* OR SU arthroplast OR (TI replace* OR AB replace* OR SU replace*) OR (TI prosthes* OR AB prosthes* OR SU prosthes*)  #5 (TI “prediction model” OR AB “prediction model” OR SU “prediction model) OR (TI predict* OR AB predict* OR SU predict*) OR (TI progn* OR AB progn* OR SU progn*) OR (TI risk prediction”OR AB risk prediction”OR SU risk prediction”) OR (TI“risk score” OR AB“risk score”OR SU“risk score”) OR (TI“risk calculation”OR AB“risk calculation”OR SU“risk calculation”) OR (TI“risk assessment”OR AB“risk assessment”OR SU“risk assessment”) OR (TI“c statistic OR AB“c statistic OR SU“c statistic) OR (TI discrimination OR AB discrimination OR SU d discrimination) OR (TI calibration OR AB calibration OR SU calibration) OR (TI auc OR AB auc OR SU auc) OR (TI “area under the curve” OR AB area under the curve” OR SU “area under the curve”) OR (TI “area under the receiver operator characteristic curve” OR AB “area under the receiver operator characteristic curve” OR SU “area under the receiver operator characteristic curve”)  #6 (((#1 AND #2) OR #3) AND #4) and #5 |

**Supplementary Table S4. PROBAST- Prediction model Risk Of Bias ASsessment Tool**

*Note: Four key domains are judged for risk of bias (low, high or unclear) and includes signalling questions to help make judgements. Signalling questions are rated as yes (Y), probably yes (PY), probably no (PN), no (N) or no information (NI), and “yes” indicates absence of bias, “no” or “probably no” flags the potential for bias. The reviewers make their judgement to determine whether the domain should be rated as “high”, “low” or “unclear” risk of bias. The first three domains are also rated for concerns regarding applicability (low/ high/ unclear).*

| **DOMAIN 1: Participants** | | | |
| --- | --- | --- | --- |
| **A. Risk of Bias** | | | |
|  | | Dev | Val |
| - 1. Were appropriate data sources used, e.g. cohort, RCT or nested case-control study data? | |  |  |
| - 1. Were all inclusions and exclusions of participants appropriate? | |  |  |
| **Risk of bias introduced by selection of participants** | **RISK:**  *(low/ high/ unclear)* |  |  |
| *Rationale of bias rating:* | | | |
|  | | | |
| **B. Applicability** | | | |
| **Concern that the included participants and setting do not match the review question** | **CONCERN:**  *(low/ high/ unclear)* |  |  |
| *Rationale of applicability rating:* | | | |
|  | | | |

| **DOMAIN 2: Predictors** | | | |
| --- | --- | --- | --- |
| **A. Risk of Bias** | | | |
|  | | Dev | Val |
| - 1. Were predictors defined and assessed in a similar way for all participants? | |  |  |
| - 1. Were predictor assessments made without knowledge of outcome data? | |  |  |
| - 1. Are all predictors available at the time the model is intended to be used? | |  |  |
| **Risk of bias introduced by predictors or their assessment** | **RISK:**  *(low/ high/ unclear)* |  |  |
| *Rationale of bias rating:* | | | |
| **B. Applicability** | | | |
| Concern that the definition, assessment or timing of predictors in the model do not match the review question | **CONCERN:**  *(low/ high/ unclear)* |  |  |
| *Rationale of applicability rating:* | | | |

| **DOMAIN 3: Outcome** | | | |
| --- | --- | --- | --- |
| **A. Risk of Bias** | | | |
|  | | Dev | Val |
| - 1. Was the outcome determined appropriately? | |  |  |
| - 1. Was a pre-specified or standard outcome definition used? | |  |  |
| - 1. Were predictors excluded from the outcome definition? | |  |  |
| - 1. Was the outcome defined and determined in a similar way for all participants? | |  |  |
| - 1. Was the outcome determined without knowledge of predictors information? | |  |  |
| - 1. Was the time interval between predictor assessment and outcome determination appropriate? | |  |  |
| **Risk of bias introduced by predictors or their assessment** | **RISK:**  *(low/ high/ unclear)* |  |  |
| *Rationale of bias rating:* | | | |
| **B. Applicability** | | | |
| Concern that the definition, assessment or timing of predictors in the model do not match the review question | **CONCERN:**  *(low/ high/ unclear)* |  |  |
| *Rationale of applicability rating:* | | | |

| **DOMAIN 4: Analysis** | | | |
| --- | --- | --- | --- |
| **Risk of Bias** | | | |
|  | | Dev | Val |
| - 1. Were there a reasonable number of participants with the outcome? | |  |  |
| - 1. Were continuous and categorical predictors handled appropriately? | |  |  |
| - 1. Were all enrolled participants included in the analysis? | |  |  |
| - 1. Were participants with missing data handled appropriately? | |  |  |
| - 1. Was selection of predictors based on univariable analysis avoided? | |  |  |
| - 1. Were complexities in the data (e.g. censoring, competing risks, sampling of controls) accounted for appropriately? | |  |  |
| - 1. Were relevant model performance measures evaluated appropriately? | |  |  |
| - 1. Were model overfitting and optimism in model performance accounted for? | |  |  |
| - 1. Do predictors and their assigned weights in the final model correspond to the results from multivariable analysis? | |  |  |
| **Risk of bias introduced by the analysis** | **RISK:**  *(low/ high/ unclear)* |  |  |
| *Rationale of bias rating:* | | | |

**Overall assessment**

| **Overall judgement about risk of bias and applicability of the prediction model evaluation** | | |
| --- | --- | --- |
| **Overall judgement of risk of bias** | **RISK:**  *(low/ high/ unclear)* |  |
| *Summary of sources of potential bias:* | | |
| **Overall judgement of applicability** | **CONCERN:**  *(low/ high/ unclear)* |  |
| *Summary of applicability concerns:* | | |
